# Preoperative Attention/Memory Problem Affects the Quality of Life of Parkinson's Disease Patients after Deep Brain Stimulation: A Cohort Study

**DOI:** 10.1155/2024/3651705

**Published:** 2024-02-07

**Authors:** Ying Gao, Jue Wang, Linbin Wang, Dianyou Li, Bomin Sun, Xian Qiu

**Affiliations:** ^1^Department of Nursing, Ruijin Hospital, Shanghai Jiao Tong University, Shanghai, China; ^2^Public Health Department, Hongqiao International Institute of Medicine, Tongren Hospital, Shanghai Jiao Tong University School of Medicine, Shanghai, China; ^3^Institute of Health Informatics, University College London, London, UK; ^4^Department of Neurosurgery, Ruijin Hospital, Shanghai Jiao Tong University School of Medicine, Shanghai, China

## Abstract

**Objectives:**

The aim of this study was to investigate the impact of nonmotor symptoms (NMS) on the quality of life (QoL) outcome after subthalamic nucleus deep brain stimulation (STN-DBS) at the 1-year follow-up.

**Methods:**

Ninety-three patients diagnosed with Parkinson's disease (PD), who underwent subthalamic nucleus deep brain stimulation (STN-DBS) between April 2020 and August 2021, were included in this study. Demographic information was gathered through a self-designed questionnaire. The severity of both motor and non-motor symptoms, along with the quality of life (QoL), was assessed using the Unified Parkinson's Disease Rating Scale-III (UPDRS-III), Nonmotor Symptoms Scale (NMSS), and 8-item Parkinson's Disease Questionnaire (PDQ-8), respectively.

**Results:**

Significant differences were observed in the UPDRS-III score, NMSS summary index (SI), and subscores of six domains (sleep/fatigue, mood/cognition, perceptual problems/hallucinations, attention/memory, urinary, and sexual function) between the baseline and the 6- and 12-month follow-ups. The correlation analysis revealed positive correlations between the preoperative NMSS SI and subscores of seven domains (cardiovascular, sleep/fatigue, mood/cognition, perceptual problems/hallucinations, attention/memory, gastrointestinal, and urinary) and ΔPDQ-8. Moreover, the preoperative PDQ-8 SI (*β* = 0.869, *P* < 0.001) and the preoperative attention/memory subscore (*β* = −0.154, *P* = 0.026) were predictive of the postsurgery improvement in quality of life (QoL).

**Conclusion:**

Deep brain stimulation (DBS) led to an improvement in the patients' nonmotor symptoms (NMS) at the 1-year follow-up, along with a correlation observed between NMS and the patients' quality of life (QoL). Notably, the severity of preoperative attention/memory problems emerged as the most significant predictor of NMS influencing the QoL outcome after STN-DBS at the 1-year follow-up.

## 1. Introduction

Parkinson's disease (PD) stands as the second most prevalent neurodegenerative condition worldwide, impacting approximately 2-3% of the population aged over 65 [1]. Alongside motor symptoms such as tremors, rigidity, and bradykinesia, PD also gives rise to a range of nonmotor symptoms (NMS). Current evidence underscores the crucial influence of NMS on the quality of life (QoL) of individuals with PD [1–3], emphasizing the importance of effective NMS management.

Deep brain stimulation of the subthalamic nucleus (STN-DBS) has been demonstrated to alleviate motor symptoms, influencing various nonmotor symptoms (NMS) to varying degrees, and enhancing the quality of life (QoL) of individuals with PD [3–6]. Nevertheless, the substantial variability in the response to DBS among patients presents a significant challenge, underscoring the utmost importance of identifying preoperative factors that can predict QoL outcomes.

Prior studies have indicated the clinical significance of nonmotor symptoms (NMS) in relation to the quality of life (QoL) and have identified NMS as a primary predictor of QoL enhancement after STN-DBS [7, 8]. However, these studies exhibited considerable heterogeneity among NMS items, thereby lacking external validity.

In this cohort study, our objective was to examine the nonmotor symptom (NMS) predictors that influence the quality of life (QoL) outcomes after STN-DBS at the 1-year follow-up. We considered the NMS domains as factors in our analysis to potentially mitigate the impact of interindividual variability while maximizing the clinical relevance of predictors for the management of Parkinson's disease (PD).

## 2. Materials and Methods

### 2.1. Patients and Study Design

A total of 160 Parkinson's disease (PD) patients who underwent subthalamic nucleus deep brain stimulation (STN-DBS) at the Department of Functional Neurosurgery in Ruijin Hospital, Shanghai, China, between April 2020 and August 2021 were enrolled in this study. From this group, 93 patients completed a 1-year follow-up and were finally included in this retrospective study. The inclusion criteria required a clinical diagnosis of PD and prior STN-DBS treatment. The exclusion criteria encompassed mental retardation, organic mental disorder, drug abuse, inability to complete follow-up, surgical complications higher than grade I [9], and difficulties understanding the questionnaires and scales at any stage of the study. All cognitive and psychiatric disorder diagnoses were made by experienced neurologists and psychiatrists. The patients underwent preoperative assessments, and further evaluations were performed at 6 and 12 months postoperatively. The study protocol received approval from the ethics committee, and written informed consent was obtained from all participating patients.

### 2.2. Clinical Assessment

Demographic and disease-related information was gathered through a self-designed questionnaire, which included age, gender, disease duration, levodopa equivalent daily doses (LEDD), Hoehn–Yahr stage (H–Y stage), and the current status of motor symptoms.

The quality of life (QoL) was evaluated using the short-form 8-item Parkinson's Disease Questionnaire (PDQ-8). Each item in the PDQ-8 represents a life situation with low quality and is rated on a 5-point Likert scale. The summary score ranges from 0 to 32 points, with higher scores indicating lower QoL. The questionnaire demonstrates good internal consistency, with a Cronbach's *α* of 0.874.

Nonmotor symptoms (NMS) were evaluated using the Nonmotor Symptoms Scale (NMSS). This scale effectively measures the severity and frequency of each nonmotor symptom and comprises 9 domains encompassing a total of 30 items. The domains include cardiovascular (2 items), sleep/fatigue (4 items), mood/cognition (6 items), perceptual problems/hallucinations (3 items), attention/memory (3 items), gastrointestinal (3 items), urinary (3 items), sexual function (2 items), and miscellaneous (4 items). Each item requires separate ratings for both frequency (1–4) and severity (0–3) of the subject's NMS. A higher score indicates more severe nonmotor symptoms.

Motor symptoms were evaluated using the Unified Parkinson's Disease Rating Scale-III (UPDRS-III). This scale, which is a subscale of the Unified Parkinson's Disease Rating Scale (UPDRS), encompasses four categories of symptoms: tremor, rigidity, bradykinesia, and axial symptoms. The total score on this scale ranges from 0 (indicating no impairment) to 108 (representing maximum impairment).

### 2.3. Statistical Analysis

Differences between the baseline scores (*x*1) and the scores at the 12-month follow-up (*x*2) after STN-DBS were calculated for each outcome parameter (Δ*x* = *x*2 − *x*1). To assess the difference in outcome parameters for each stage, we employed a one-way analysis of variance (ANOVA). Subsequent post hoc comparisons were conducted using Tamhane's T2 test. The *p* value was corrected using the Bonferroni correction method. When appropriate, independent samples *t*-test or Mann–Whitney *U* test were used to compare subgroups. Pearson's correlation analysis was performed between the outcome parameters. To predict the postoperative improvement in quality of life (QoL), we conducted multivariate linear regression, with ΔPDQ-8 as the dependent variable, and the factors found significant in Pearson's correlation analysis as independent variables, while also controlling for sex and age. The statistical analysis was carried out using SPSS version 20 (SPSS, Inc., Chicago, IL, USA), with the significance level set at 0.05.

## 3. Results

### 3.1. Demographic Information

Ninety-three patients diagnosed with Parkinson's disease (PD) were enrolled in this study. Demographic and disease-related information is presented in Table 1. All the information of patients was obtained during their off conditions. Additional specific characteristics can be found in Supplementary Table 1.

### 3.2. The Effects of STN-DBS on NMS and QoL

**Figure 1:**
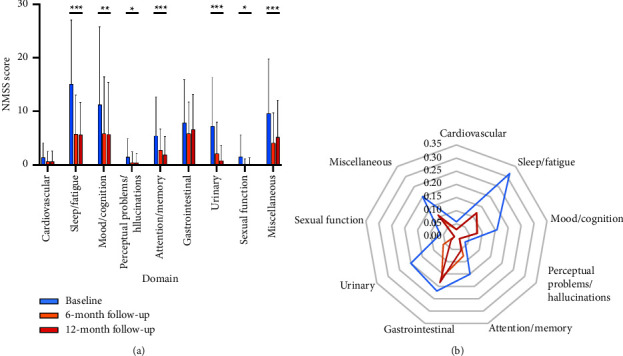
NMSS domains at baseline (blue), 6-month follow-up (orange), and 36-month follow-up (red) in a bar chart (a) and radar chart (b). (a) Sleep/fatigue, mood/cognition, perceptual problems/hallucinations, attention/memory, and urinary domain significantly improved at both 6- and 12-month follow-ups compared to the baseline. Sexual function and miscellaneous domain showed significant improvement at 6-month follow-up. However, the sexual function domain stayed unchanged at 12-month follow-up, while the miscellaneous domain worsened significantly at the 12-month follow-up. (b) NMSS domain scores were illustrated as percentages of each maximum score. The NMS burden was overall mitigated at both 6- and 12-month follow-ups compared to the baseline. ^*∗*^indicates *P* < 0.05, ^*∗∗*^indicates *P* < 0.01, ^*∗∗∗*^indicates *P* < 0.001.

To investigate the impact of DBS on patients' nonmotor symptoms (NMS) and quality of life (QoL), we compared the PDQ-8 index and NMSS score across nine domains at three time points: before DBS and at 6- and 12-month follow-ups. Univariate analysis (Table 2 and Figure 1) revealed a significant difference in the PDQ-8 index and in the scores for the sleep/fatigue, mood/cognition, perceptual problems/hallucinations, attention/memory, urinary, and sexual function domains, as well as the NMSS total score. Post hoc tests were conducted to further elucidate the significant differences observed in the variables at the three time points.

### 3.3. The Relationship between NMS and QoL Improvement

As previously mentioned, there were significant differences in PDQ-8 indexes at each time point. ΔPDQ-8 was used to represent the changes in quality of life (ΔPDQ-8 = PDQ-8_baseline_ − PDQ-8_12M_). Correlation analysis was performed to examine the association between ΔPDQ-8 and various demographic and disease factors, including age, gender, disease duration, levodopa equivalent daily doses (LEDD), UPDRS-III score, Hoehn–Yahr level (H–Y level), preoperative NMSS score, and PDQ-8 index before DBS. The results, as shown in Table 3, revealed positive correlations between ΔPDQ-8 and the preoperative PDQ-8 index, as well as seven subscores of NMSS before DBS (cardiovascular, sleep/fatigue, mood/cognition, perceptual problems/hallucinations, attention/memory, gastrointestinal, and urinary), along with the preoperative NMSS total score.

### 3.4. The Nonmotor Predictors for QoL Improvement after STN-DBS

Multivariate linear regression analysis was conducted to investigate the predictors of quality of life (QoL) improvement following STN-DBS. Based on the regression model, the preoperative PDQ-8 index (*β* = 0.867, *P* < 0.001) and the preoperative attention/memory score (*β* = −0.158, *P*=0.024) were found to be significant predictors of QoL improvement at the 12-month follow-up (Table 4).

## 4. Discussion

In this retrospective cohort study, we investigated the impact of STN-DBS on nonmotor symptoms (NMS) and quality of life (QoL), while also identifying the correlation between these two parameters. The findings indicated that DBS led to an improvement in patients' NMS at the 1-year follow-up, and a correlation was observed between NMS and patients' QoL. Notably, the severity of preoperative attention/memory problems emerged as the most prominent predictor of NMS influencing the QoL outcome after STN-DBS at the 1-year follow-up.

### 4.1. Nonmotor Symptoms

STN-DBS showed significant relief in the burden of nonmotor symptoms (NMS) and improvement in patients' quality of life (QoL), which is in line with the findings of Dafsari et al. [10, 11]. Notably, seven domains of NMSS exhibited significant improvement at both the 6- and 12-month follow-ups when compared to the baseline.

#### 4.1.1. Sleep/Fatigue

Previous studies have shown a significant improvement in sleep quality, efficiency, and duration after STN-DBS [12]. Consistent with these findings, our study also observed a significant difference in the NMSS sleep/fatigue domain at the 6-month follow-up [10, 13]. Although the degree of improvement decreased, the sleep/fatigue domain showed significant enhancement at the 12-month follow-up compared to the 6-month follow-up. Physiologically, dopaminergic neurons in the ventral tegmental area of the midbrain are regulated by orexin neurons from the hypothalamus, forming a descending loop that influences arousal and wakefulness from the cortex and thalamus to the pontine nuclei and reticular formation [14]. Subthalamic nucleus (STN) neurons have neural projections in regulatory regions, such as the cortex, thalamus, and pedunculopontine nucleus, which are involved in sleep regulation. Therefore, STN may be part of the regulatory networks governing sleep and arousal functions, potentially influencing sleep disorders [15]. The observed improved sleep after STN-DBS in our patients may also be associated with the alleviation of nocturnal dyskinesia, reduced nocturia, and pain relief. Additionally, as sleep disturbances in PD patients contribute to fatigue, the improvement in sleep may also lead to the relief of fatigue in these patients [16].

#### 4.1.2. Mood/Cognition

In the current study, we observed significant differences in the NMSS mood/cognition domain at both the 6- and 12-month follow-ups, which diverged from some previous findings [10, 17]. Nevertheless, our results were consistent with several other studies. For instance, Elizabeth et al. reported that unilateral STN-DBS led to improved depression in patients at the 6-month follow-up [18], and similar findings were reported by Li et al. [19]. A systematic review by Couto et al. also demonstrated that depressive symptoms in PD patients showed improvement within the initial months after STN-DBS [20]. Additionally, about 40% of PD patients experience anxiety, and STN-DBS has shown some degree of improvement in this condition. However, apathy, which arises due to the downregulation of dopamine modulation in the limbic basal ganglia, may worsen after STN-DBS due to a reduction in dopaminergic medication use [21]. A recent study indicated that STN-DBS has a short-term (<3 months) beneficial effect on apathy, but in the long term, it could exacerbate the condition [22].

#### 4.1.3. Perceptual Problems/Hallucinations

Our observations indicated a significant improvement in the NMSS perceptual problems/hallucinations domain at both the 6- and 12-month follow-ups, which is in agreement with the findings of Dafsari et al. and Yoshida et al. [10, 23]. However, Jost et al. did not report any significant change in this domain from baseline to the 36-month follow-up [17]. Due to the limited number of studies focusing on this specific issue, further research is required to elucidate the correlation between STN-DBS and outcomes related to perceptual problems or hallucinations.

#### 4.1.4. Attention/Memory

In our study, the NMSS attention/memory domain demonstrated a significant improvement at both the 6- and 12-month follow-ups. However, it is essential to verify this result as existing studies have not consistently reported any significant change in attention or memory in PD patients after STN-DBS. For instance, Jost et al. found no improvement in the attention/memory domain at the 36-month follow-up [17]. Similarly, Funkiewiez et al. reported no significant change in cognitive functions, attention, and memory three years after surgery [24]. Eghlidos's meta-analysis also supported this conclusion, suggesting that STN-DBS does not significantly improve memory, or there is only a slight improvement in patients [25, 26]. Additionally, according to Kim et al., there is no evidence to suggest that STN-DBS is a risk factor for cognitive deterioration in PD patients when compared to medical therapy [27]. Moreover, only 3 items exist in the NMSS evaluating patient's attention and memory function, which demonstrates that more systematic tools are needed to assess patient's attention and memory function.

#### 4.1.5. Urinary

Consistent with a prospective study by Dafsari et al., we observed a significant improvement in the NMSS urinary domain at the 6-month follow-up [10]. Additionally, Herzog et al. reported that subthalamic stimulation modulates the cortical control of the urinary bladder, leading to an amelioration of bladder dysfunction in PD patients. This improvement may be attributed to the “normalization” of neural activity in the network responsible for cerebral bladder control, coupled with lateral frontal cortex activation [28]. Furthermore, this improvement was sustained at the 12-month follow-up, aligning with the findings of the long-term study conducted by Jost et al. [17].

#### 4.1.6. Sexual Function

In our study, we observed a significant improvement in the NMSS sexual function domain at the 6-month follow-up. This finding aligns with the results of a study by Castelli et al., which involved a cohort of 31 PD patients (21 males and 10 females) [29]. Another study showed that 21 male patients reported significant improvements in their sexual life after STN-DBS. However, in our study, the average level of sexual function at the 12-month follow-up remained similar to that at the 6-month follow-up. On the other hand, Jost et al. only reported a significant difference in sexual function at the 36-month follow-up compared to baseline, but the changing trend of sexual function during the 36-month follow-up was not reported [17]. Therefore, we concluded that the effect of STN-DBS on PD patients' sexual function was mainly concentrated in the short postoperative period.

#### 4.1.7. Miscellaneous

In our study, the NMSS miscellaneous domain, which includes pain, abnormal olfaction, altered weight, and hyperhidrosis, demonstrated a significant improvement at the 6-month follow-up, consistent with the findings of Jost et al. [17]. Surprisingly, at the 12-month follow-up, the miscellaneous domain deteriorated significantly compared to the 6-month follow-up. However, the miscellaneous burden at the 12-month follow-up was still significantly relieved compared to baseline, indicating an unstable long-term effect of STN-DBS on the miscellaneous domain. Furthermore, previous studies have reported varied results regarding specific components of the miscellaneous domain. For instance, Wolz et al. found no immediate improvement in excessive sweating and pain after STN-DBS [30]. In contrast, Cury et al. demonstrated that STN-DBS surgery improved patients' pain [31]. The effects of STN-DBS on olfaction may be mediated through modulation of the orbitofrontal and primary olfactory cortices, and Kola et al. found deterioration of olfaction in patients after STN-DBS [32]. Additionally, Strowd et al. reported weight gain in PD patients after STN-DBS [33]. In the study by Bjerknes et al., the trends of autonomic symptoms after STN-DBS were similar to our present study, initially showing significant improvement and then worsening over time. Autonomic symptoms in PD patients tend to worsen with age and disease progression [34]. Contrary to the observed improvement in the miscellaneous domain, we found no significant improvement in the cardiovascular and gastrointestinal domains at either the 6- or 12-month follow-up.

#### 4.1.8. Cardiovascular

In our study, the NMSS cardiovascular domain remained unchanged at both the 6- and 12-month follow-ups, which aligns with the findings of Dafsari et al. [10]. Similarly, Wolz et al. did not report any immediate improvement in dizziness after STN-DBS [30]. Although no significant difference was observed, the average level of the cardiovascular domain slightly worsened at the 12-month follow-up compared to the 6-month follow-up. Thus, we concluded that STN-DBS has little influence on cardiovascular symptoms.

#### 4.1.9. Gastrointestinal

In our study, the NMSS gastrointestinal domain showed no significant differences at both the 6- and 12-month follow-ups. This result was consistent with the findings of Dafsari et al. and Jost et al. [10, 17]. Gastrointestinal symptoms in PD may be caused by extracerebral lesions in patients, such as dysphagia which may be due to muscle wasting, abnormal peristalsis, or esophageal contractions [35]. However, Arai et al. demonstrated that STN-DBS improves gastric emptying dysfunction in PD patients, and this phenomenon might be caused by altering the neural system controlling gastrointestinal function. Although the underlying mechanism was unclear, the activation of nerve fibers projecting from or to the hypothalamus and crossing the subthalamic nucleus was a possible route [36]. Wolz et al. reported that dysphagia in 34 PD patients significantly improved after STN-DBS [30].

### 4.2. QoL

The PDQ-8 summary index improved substantially at the 6- and 12-month follow-ups, which was consistent with the studies by Dafsari et al. and Jost et al. [10, 17]. To investigate the degree of QoL improvement and its influencing factors, we created the variable ΔPDQ-8 to quantify the change in the PDQ-8 summary index. The correlation analysis between ΔPDQ-8 and factors revealed significant correlations with the UPDRS-III score, NMSS total score, and preoperative PDQ-8 summary index. The correlation with the NMSS total score was stronger than the UPDRS-III score, indicating that nonmotor burden plays a critical role in PD patients' QoL. This finding was consistent with the previous study and highlighted the importance of NMS in patients' response to STN-DBS [1, 37]. Specifically, ΔPDQ-8 significantly correlated with seven preoperative NMSS domains: cardiovascular, sleep/fatigue, mood/cognition, perceptual problems/hallucinations, attention/memory, gastrointestinal, and urinary. This observation suggested that even if a specific domain of NMSS showed no significant improvement from STN-DBS, these symptoms would still impact the change in QoL and patients' overall benefit from the surgery.

### 4.3. Predictors of QoL Improvement

Existing evidence has demonstrated that the severity change of motor symptoms is the most significant predictive factor for the improvement of PD patients' QoL [38]. On the other hand, although NMS does not directly affect patients' motor function, it can significantly impact their QoL. Therefore, as a common clinical goal to alleviate the NMS burden, we should consider the extent of QoL improvement for patients undergoing DBS in the future. In this study, we constructed a regression model to predict the improvement of patients' QoL based on correlated variables. To the best of our knowledge, this is one of the few studies that predicts PD patients' QoL improvement from STN-DBS based on preoperative characteristics. The regression model revealed that the preoperative NMSS attention/memory domain and the preoperative PDQ-8 summary index could predict the QoL benefit from STN-DBS. The preoperative attention/memory domain showed a negative correlation with ΔPDQ-8, indicating that PD patients with mild attention/memory symptoms tend to experience marked improvement in QoL from STN-DBS. This finding suggests that the decline in cognition, memory, and attention in patients is most likely a result of disease progression. A lower level of cognition at baseline indicates a smaller degree of QoL improvement following DBS surgery and less benefit from the procedure. Studies by Rački et al. have suggested that surgery and electrical stimulation of DBS may have the potential to cause some degree of brain damage. Although no significant improvement was detected in attention/memory levels over 1 year, as indicated by the results, patients with poor preoperative cognition may have been significantly affected by surgery, leading to less gain in QoL [39]. Meanwhile, using only the entries in NMSS to evaluate attention and memory function is not comprehensive enough. Therefore, the conclusions about attention and memory function based on NMSS may have certain limitations. Similarly, Witt et al. reported that the poor postoperative improvement in QoL of PD patients with borderline cognitive levels was likely correlated with their low cognitive function [40]. In addition to directly affecting patients' self-judgment of quality of life, preoperative cognitive function level may also amplify patients' comparative feelings before and after STN-DBS, thereby affecting patients' self-reported quality of life benefits. In conclusion, these findings underscore the importance of conducting a cognitive assessment before DBS.

Conversely, the preoperative PDQ-8 summary index positively correlated with ΔPDQ-8, suggesting that PD patients with a low preoperative QoL experienced more significant improvement in QoL after STN-DBS. This finding aligns with the results of Jost et al. [7]. Daniels et al. also reported that the change in PD patients' QoL had correlations with preoperative parameters, including positive mood changes, which did not show a significant correlation in our study [38]. This could be related to the different scales evaluating patients' mood used in Daniels' study and this one. Thus, gaining an in-depth understanding of the type of PD patient who would benefit most from STN-DBS and elucidating the mechanisms by which STN-DBS improves NMS would be valuable. It would also aid physicians and surgeons in predicting the benefits for PD patients after STN-DBS and providing more precise management recommendations.

### 4.4. Limitations

Nevertheless, the present study has several limitations. First, subgroup analysis was not performed in the univariate analysis of QoL and NMS. The changes in specific domains of NMSS after STN-DBS may be influenced by other factors, such as age, gender, and disease duration. Subgroup analysis could help elucidate significant factors by comparing the target parameters of different subgroups divided by a specific potential factor. Therefore, the current study cannot provide further insights into the postoperative changes in each NMS. Second, considering the advanced age of our cohort, the scale questions in the NMSS sex function domain were difficult to answer or were not fully applicable to PD patients. This discrepancy between the scale and the cohort might have led to low-quality data and may have influenced the study results. Third, the samples in our study were from a single center, which could introduce a systematic bias. Thus, future studies involving multiple centers with larger sample sizes, multiple evaluation tools, and exhaustive data analysis are anticipated. Fourth, this study lacks further evaluation of patient attention and memory function. In the future, more comprehensive tools are needed for detailed evaluation. Lastly, new onset impulse control disorders were not evaluated in the samples of this study. Given the potential impact of new-onset impulse control disorders on NMS and patients' QoL, we look forward to further consideration of these disorders in future related studies.

## 5. Conclusions

This retrospective cohort study with 6- and 12-month follow-ups provides evidence for the improvement of QoL and NMS burden by STN-DBS. We demonstrated significant improvement in seven domains of NMSS at both 6- and 12-month follow-ups: sleep/fatigue, mood/cognition, perceptual problems/hallucinations, attention/memory, urinary, sex function, and miscellaneous. Apart from the total NMS burden and preoperative QoL, the improvement in QoL through STN-DBS was significantly correlated with the cardiovascular, sleep/fatigue, mood/cognition, perceptual problems/hallucinations, attention/memory, gastrointestinal, and urinary domains. Moreover, the improvement in QoL could be predicted by the preoperative attention/memory domain and preoperative QoL, which might aid in the prediction of prognosis and clinical management of patients.

## Figures and Tables

**Table 1:** Demographic and disease information (*N* = 93).

Variable	Group	$\underset{¯}{x}\pm s$ /number (%)
Age	—	62.94 ± 9.68

Disease duration (year)	—	9.61 ± 4.31

LEDD (mg/day)	—	553.76 ± 307.72

UPDRS-III	—	59.24 ± 11.82

Gender	Male	55 (59.1)
	Female	38 (40.9)

H–Y stage	2.0	11 (11.8)
	2.5	23 (24.8)
	3.0	43 (46.2)
	4.0	16 (17.2)

LEDD: levodopa equivalent daily doses and H–Y stage: Hoehn–Yahr stage.

**Table 2:** Outcome parameters assessed at preoperative baseline and at postoperative 6- and 12-month follow-ups for a total of 93 participants (*N* = 93).

Variable	Baseline		6-month follow-up		12-month follow-up		*P*	Post hoc tests
	Mean	SD	Mean	SD	Mean	SD		
PDQ-8 summary index^*∗∗∗*^	12.42	6.04	3.92	4.04	3.03	3.86	<0.001	a b c
NMSS domains								
Cardiovascular	1.39	2.67	0.67	1.86	0.69	1.91	0.075	—
Sleep/fatigue^*∗∗∗*^	15.12	11.95	5.77	7.27	5.67	6.03	<0.001	a b c
Mood/cognition^*∗∗*^	11.27	14.57	5.86	10.62	5.70	9.73	0.006	a b c
Perceptual problems/hallucinations^*∗*^	1.47	3.47	0.47	1.96	0.43	1.68	0.030	a b c
Attention/memory^*∗∗∗*^	5.43	7.23	2.77	3.94	1.91	3.43	<0.001	a b c
Gastrointestinal	7.87	8.04	5.89	5.86	6.63	6.58	0.163	—
Urinary^*∗∗∗*^	7.23	9.17	2.10	5.93	0.85	2.79	<0.001	a b c
Sexual function^*∗*^	1.51	4.06	0.18	0.99	0.18	1.17	0.010	a b c
Miscellaneous^*∗∗∗*^	9.63	10.14	4.08	5.66	5.19	6.88	<0.001	a b c
NMSS total score^*∗∗∗*^	60.91	47.98	27.80	26.68	27.26	25.32	<0.001	a b c

Post hoc comparisons (Tamhane's T2 test): a = significant difference between baseline and 6-month follow-up (*P* < 0.05); b = significant difference between baseline and 12-month follow-up (*P* < 0.05); c = significant difference between 6-month follow-up and 12-month follow-up (*P* < 0.05). ^*∗*^indicates *P* < 0.05, ^*∗∗*^indicates *P* < 0.01, ^*∗∗∗*^indicates *P* < 0.001.

**Table 3:** The correlations between the test scores at the preoperative baseline and the 12-month change scores of quality of life (*N* = 93).

	PDQ-8 SI change score	
	*r*	*P*
PDQ-8 SI	0.80^*∗∗∗*^	<0.001
Baseline NMSS domains		
Cardiovascular	0.22^*∗*^	0.032
Sleep/fatigue	0.33^*∗∗*^	0.001
Mood/cognition	0.49^*∗∗∗*^	<0.001
Perceptual problems/hallucinations	0.36^*∗∗∗*^	<0.001
Attention/memory	0.22^*∗*^	0.031
Gastrointestinal	0.36^*∗∗∗*^	<0.001
Urinary	0.27^*∗*^	0.010
Sexual function	0.11	0.291
Miscellaneous	0.18	0.090
NMSS total score	0.46^*∗∗∗*^	<0.001

^*∗*^indicates *P* < 0.05, ^*∗∗*^indicates *P* < 0.01, ^*∗∗∗*^indicates *P* < 0.001.

**Table 4:** Multivariate linear regression analysis of QoL variation (*N* = 93).

Variables	*B*	SE	*β*	*t*	*P*
Constant	−0.789	0.850	—	−0.928	0.356
PDQ-8 SI_baseline_^*∗∗∗*^	0.869	0.068	0.869	12.730	<0.001
Cardiovascular_baseline_		—	0.027	0.405	0.687
Sleep/fatigue_baseline_	—	—	0.089	1.335	0.185
Mood/cognition_baseline_	—	—	0.070	0.869	0.387
Perceptual problems/hallucinations_baseline_	—	—	0.130	1.943	0.055
Attention/memory_baseline_^*∗*^	−0.130	0.057	−0.154	−2.262	0.026
Gastrointestinal_baseline_	—	—	0.056	0.832	0.407
Urinary_baseline_	—	—	0.037	0.556	0.580
NMSS total score_baseline_	—	—	0.092	1.064	0.290

*R* ^2^ = 0.662, adjusted *R*^2^ = 0.654, and D-W = 1.902. Residuals are independent. *F* = 5.115 and *P*=0.026. ^*∗*^indicates *P* < 0.05, ^*∗∗∗*^indicates *P* < 0.001.

## Data Availability

The patient data used to support the findings of this study have not been made available because of patients' privacy.

## Supplementary Materials

Supplementary Materials A total of 93 PD patients were included in this study. Demographic and disease information is displayed in Table 1 in the main text. Other specific characteristics are listed in Supplementary Table 1.
