# Supplementary material for: Preoperative Attention/Memory Problem Affects the Quality of Life of Parkinson's Disease Patients after Deep Brain Stimulation: A Cohort Study
Source: Parkinsons Dis. 2024 Feb 7;2024:3651705. doi: 10.1155/2024/3651705 (PMC10866634; doi:10.1155/2024/3651705)
Supplement: Supplementary Materials — A total of 93 PD patients were included in this study. Demographic and disease information is displayed in Table 1 in the main text. Other specific characteristics are listed in Supplementary Table 1. [file 3651705.f1.docx]

Supplementary Table 1. Demographic and disease information (*N* = 93)

| **Variable** | **Group** | $\bar{\boldsymbol{x}}\boldsymbol{\pm s}$ **/ Number (%)** |
| --- | --- | --- |
| Age | - | 62.94 ± 9.68 |
| BMI | - | 23.54 ± 3.12 |
| Disease duration | - | 9.61 ± 4.31 |
| LEDD (mg/day) | - | 553.76 ± 307.72 |
| UPDRS-III | - | 59.24 ± 11.82 |
| Gender | Male | 55 (59.1) |
|  | Female | 38 (40.9) |
| Residence | Rural area | 26 (27.9) |
|  | Urban area | 67 (72.0) |
| Occupation | Retired | 75 (80.6) |
|  | On duty | 11 (11.8) |
|  | Unemployed | 7 (7.5) |
| Marriage | Married | 92 (98.9) |
|  | Divorced | 1 (1.0) |
| Education | Primary school and below | 16 (17.2) |
|  | Junior high school | 21 (22.5) |
|  | High school | 28 (30.1) |
|  | Associate degree | 19 (20.4) |
|  | Undergraduate degree | 6 (6.4) |
|  | Graduate degree | 3 (3.2) |
| H-Y stage | 2.0 | 11 (11.8) |
|  | 2.5 | 23 (24.8) |
|  | 3.0 | 43 (46.2) |
|  | 4.0 | 16 (17.2) |

*BMI: Body mass index; LEDD: Levodopa equivalent daily doses; H-Y stage: Hoehn-Yahr stage*
